# Combination of Plasma Pharmacochemistry, RNA-Seq, and Molecular Docking Strategies to Reveal the Mechanism of the Alkaloid Fraction of *Nelumbinis folium* for the Treatment of Hyperlipidemia

**DOI:** 10.3390/molecules30183727

**Published:** 2025-09-12

**Authors:** Yuan Cai, Rong Huang, Tianfeng Lin, Leyi Yang, Chang Zhou, Yumiao Li, Bin Liu, Shifen Dong, Yanyan Jiang

**Affiliations:** School of Chinese Materia Medica, Beijing University of Chinese Medicine, Beijing 102488, China; cai13263208090@163.com (Y.C.); ht15286223413@163.com (R.H.); lintianfeng913@163.com (T.L.); 18811305627@163.com (L.Y.); zhouchang0226@163.com (C.Z.); liyumiao6677@163.com (Y.L.); liubinyn67@163.com (B.L.)

**Keywords:** alkaloid fraction of *Nelumbinis folium*, hyperlipidemia, plasma pharmacochemistry, transcriptome-sequencing technology, molecular docking technology

## Abstract

*Nelumbinis folium* (*N. folium*) exhibits hypolipidemic effects and shows great potential for application in lipid-lowering drugs and healthcare products. This study aimed to investigate the mechanism underlying the hypolipidemic effects of the alkaloid fraction of *N. folium* (AFN). Animal experiments demonstrated that AFN significantly reduced blood lipid levels and ameliorated liver damage in hyperlipidemic mice. RNA-seq analysis identified 26 reverse-regulated differentially expressed genes (DEGs), which were primarily involved in the PPAR signaling pathway, fat digestion and absorption, and fatty acid degradation. Using UPLC-MS^n^, 30 plasma-absorbed components were identified, including 13 prototype alkaloids. Among these, three key active components—nuciferine, *N*-nornuciferine, and *N*-methylisococlaurine—were screened via network topology analysis. Molecular docking revealed strong binding affinities between these compounds and key targets. The results showed that *N*-methylisococlaurine bound to *SLC27A4* and *CPT1A* with strong affinity, while nuciferine and *N*-nornuciferine bound to *ACADVL* and *PPARA*. RT-qPCR results confirmed that AFN modulates the expression of *FABP1*, *SLC27A4*, *PPARA*, *CPT1A*, *ACAA2*, *APOC3*, and *APOA4*. These findings suggest that AFN exerts its hypolipidemic effects through multi-component, multi-target, and multi-pathway mechanisms.

## 1. Introduction

Hyperlipidemia, a prevalent disorder of lipid metabolism with increasing incidence, is a significant risk factor for numerous conditions including hypertension, coronary heart disease, diabetes, pancreatitis, hyperuricemia, and cardiovascular diseases, severely impacting patient quality of life [1]. Current clinical management relies on chemical agents such as bile acid sequestrants, cholesterol absorption inhibitors, statins, probucol, microsomal triglyceride transfer protein inhibitors, and fibrates. However, long-term use of these drugs is often associated with adverse effects, including potential liver and kidney damage [2,3]. Consequently, there is a growing imperative to identify safer and effective therapeutic alternatives.

Traditional Chinese Medicines (TCMs) represent a promising source for such alternatives. *Nelumbinis folium* (*N. folium*), the dried leaf of *Nelumbo nucifera* Gaertn. (recorded in the Chinese Pharmacopoeia 2020), has garnered significant attention for its lipid-lowering properties. Modern pharmacological studies confirm that *N. folium* effectively regulates blood lipids, reduces weight, and inhibits fatty liver, leading to its widespread use in lipid-lowering drugs and healthcare products. Among its diverse chemical constituents (alkaloids, flavonoids, terpenes, lignans, etc.), alkaloids are recognized as the characteristic and primary bioactive components responsible for its hypolipidemic effects. Notably, preliminary in vitro studies by our group identified *O*-nornuciferine and nuciferine as key alkaloids modulating pathways such as bile secretion, glycerophospholipid and sphingolipid metabolism, and the *PPAR* signaling pathway [4], providing a foundation for investigating their mechanisms in vivo.

Despite this foundation, a comprehensive understanding of the in vivo hypolipidemic mechanism of *N. folium* alkaloids, particularly at a systemic level, remains incomplete. Fortunately, modern research techniques provide powerful tools to bridge this gap [5]. For example, network pharmacology combined with molecular docking technology elucidates that Polygonati Rhizoma acts on key targets such as *RXRA*, *PIK3CA*, and *ESR1* through a series of chemical components such as DFV, apigenin, and baicalein, tThereby exerting lipid-lowering effects [6]. Proteomics technology has been widely applied in the research of hyperlipidemia. Research shows Korean red ginseng exerts lipid-lowering effects by modulating *PCSK9* and *LDLR* expression thereby regulating cholesterol metabolism through the sterol regulatory element-binding protein 2 (*SREBP2*)/*PCSK9*/*LDLR* signaling pathway [7]. The combined analysis of multiple omics such as metabolomics and transcriptomics revealed that the lipid-lowering and liver-protecting effects of milk thistle solid beverage significantly regulated cholesterol synthesis and neolipogenesis, as well as FA-*β*-oxidation [8]. These methodologies align with the holistic perspective of TCM by facilitating the study of complex interactions between herbal compounds and the diseased organism at a systems level, making them highly suitable for elucidating the mechanisms of TCMs like *N. folium*.

To address the critical need for elucidating the in vivo mechanism of *N. folium* alkaloids, this study employed an integrated strategy combining plasma pharmacochemistry, transcriptome sequencing, UPLC-MS^n^ analysis, and molecular docking. Specifically, we focused on investigating the hypolipidemic mechanism of the Alkaloid Fraction of *N. folium* (AFN) in vivo. By synergistically applying these techniques, we aimed to deeply and systematically elucidate the mechanisms underlying the lipid-lowering effects of AFN and its active constituents.

## 2. Results

### 2.1. Content Determination of 4 Alkaloid Components

**Figure 1:**
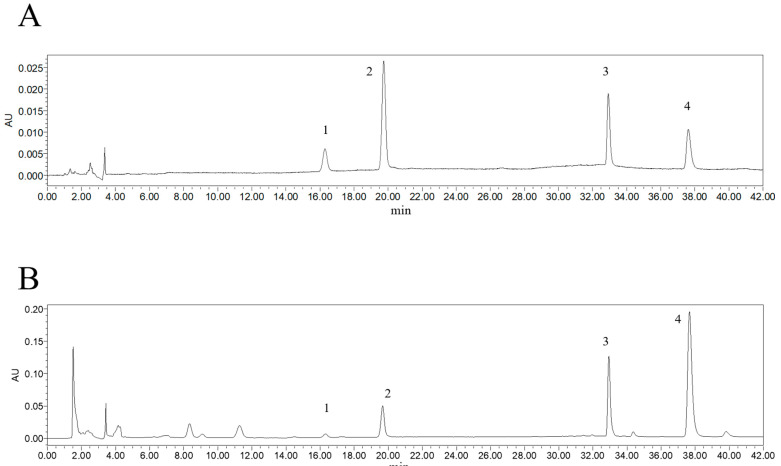
Determination of four alkaloid components in AFN. (**A**) Plot of four alkaloid components as controls under UV 285 nm conditions. (**B**) Determination results of four alkaloid components in AFN prepared from the eighth batch of *N. folium* medicinal materials under ultraviolet light at 285 nm. Peak 1 is armepavine, peak 2 is 2-hydroxy-1-methoxyaporphine, peak 3 is *N*-nornuciferine, and peak 4 is nuciferine.

Methodological evaluation: precision evaluation, stability evaluation, repeatability evaluation, accuracy evaluation, and recovery rate were all good. The results indicate that this method can be accurate and reliable. Under the above experimental conditions, the contents of four alkaloids in different *N. folium* samples were determined as follows: the contents of armepavine, 2-hydroxy-1-methoxyaporphine, *N*-nornuciferine, and nuciferine in 10 batches of *N. folium* medicinal materials were 0.65–1.90%, 1.54–6.01%, 4.21–12.00%, 10.71–56.62%, respectively (Table S5). Among them, the content of nuciferine is the highest (Figure 1). The stacked HPLC traces of 10 batches of *N. folium* is shown in Figure S1.

### 2.2. AFN Reduces Blood Lipids and Ameliorates Liver Damage in Mice

**Figure 2:**
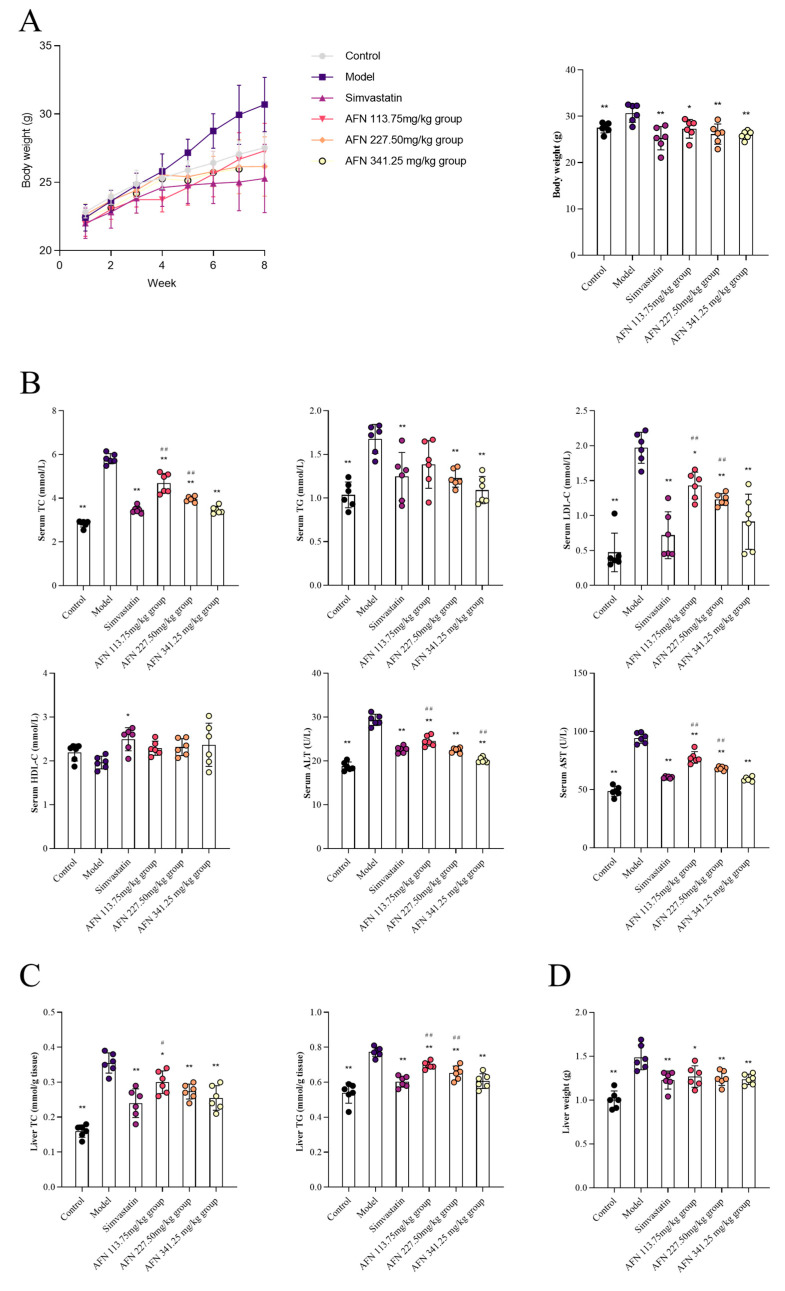
AFN reduced blood lipids and ameliorated liver damage in mice. (**A**) Weight change within 8 weeks and final body weight. (**B**) Effects of AFN on serum biochemical indices TC, TG, LDL-C, HDL-C, ALT, and AST in hyperlipidemic mice. (**C**) AST and ALT levels in liver tissues. (**D**) Weight of livers. All data are shown as the Mean ± SD (*n* = 6). * *p* < 0.05, ** *p* < 0.01 vs. the Model group. ^#^ *p* < 0.05, ^##^ *p* < 0.01 vs. the Simvastatin group.

Compared with the model group, AFN at dosages of 113.75 mg/kg, 227.50 mg/kg and 341.25 mg/kg, and simvastatin 15.17 mg/kg could significantly reduce the levels of serum TC and LDL-C (*p* < 0.01, *p* < 0.05). All three dose groups could significantly reduce the levels of ALT and AST in the serum of hyperlipidemia mice (*p* < 0.01) (Figure 2B).

After the intervention of AFN, compared with the model group, the liver weight of mice in the drug groups was significantly decreased (*p* < 0.05) (Figure 2D). Compared with the model group, the contents of TC and TG in the liver of mice in the three dose groups were significantly decreased (*p* < 0.01, *p* < 0.05), indicating that the AFN could effectively inhibit the accumulation of lipids in the liver of hyperlipidemic mice, and had a specific inhibitory effect on the occurrence and development of liver lesions such as fatty liver (Figure 2C). The mice in the dosing groups had varying degrees of weight reduction (Figure 2A).

### 2.3. AFN Regulated Gene Expression in Mice Livers

**Figure 3:**
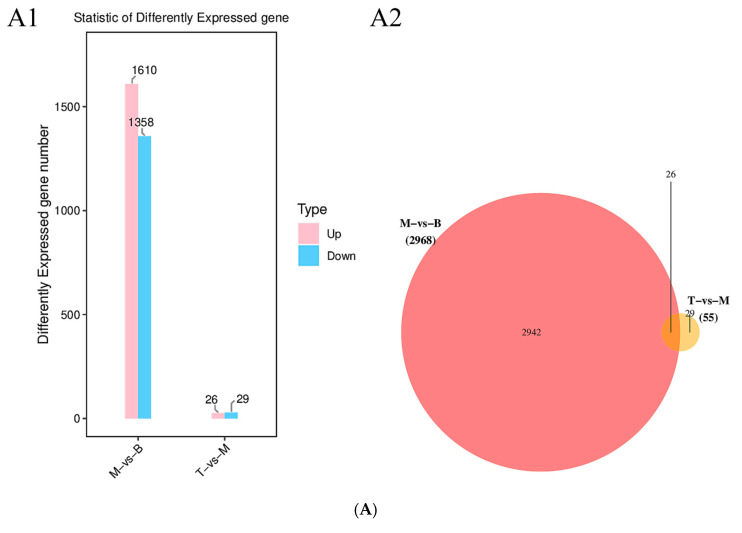

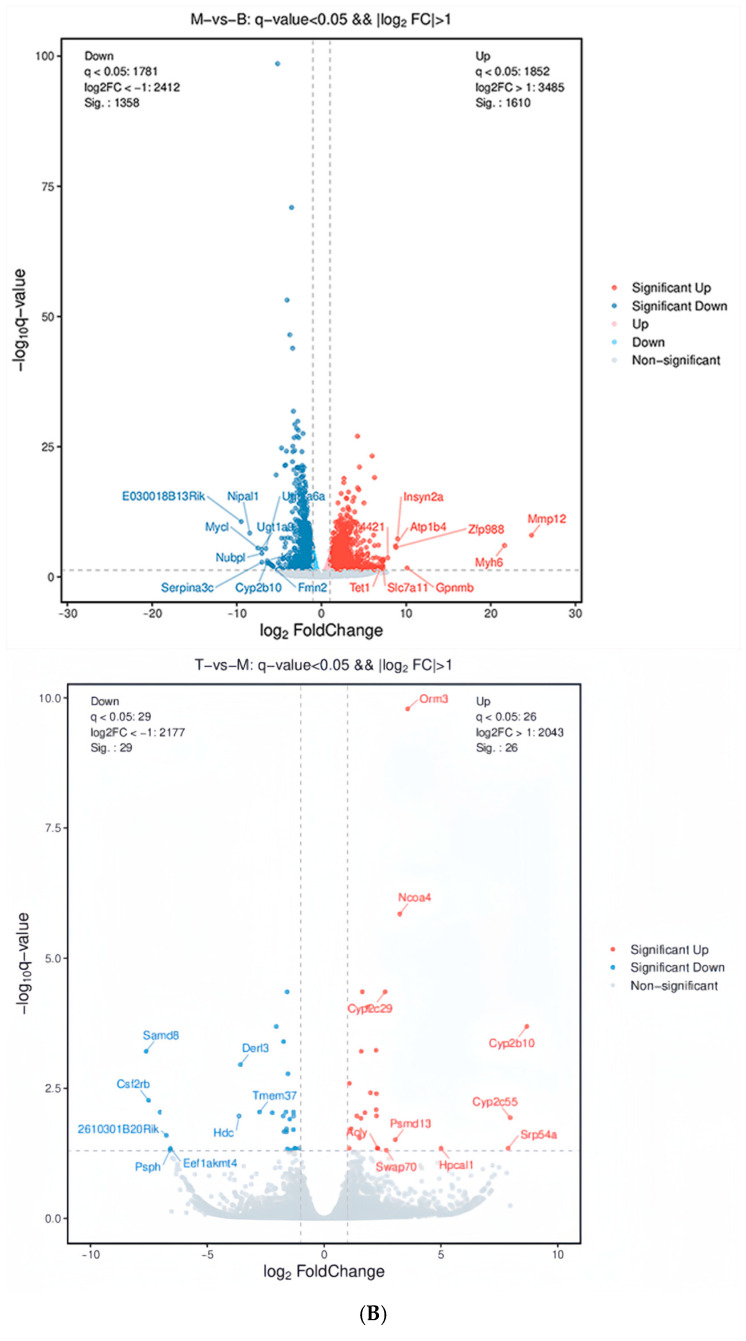
(**A**). Transcriptomic analysis of mouse liver tissues induced by AFN on high-fat diets. (**A1**) DEGs statistical plot (**A2**) Venn diagram of DEGs between groups. The threshold value was FC > 2 or FC < 0.5, and the *p*-adjust value was <0.05. (**B**). Transcriptomic analysis of mouse liver tissues induced by AFN on high-fat diets. Volcano plot of model vs. control and AFN 341.25 mg/kg group vs. Model.

In order to identify DEGs, the form of pairwise comparison between sample groups was used; that is, the control group was compared with the model group, and the AFN 341.25 mg/kg group was compared with the model group. A cut-off of *p*-value < 0.05 and ∣log2FC∣ > 1 was applied, and the significantly changed genes were shown in the volcano plot (Figure 3B). It could be seen that 1610 differentially expressed genes were up-regulated, and 1358 differentially expressed genes were down-regulated in the control group and model group. In the model group and AFN 341.25 mg/kg group, 26 genes were up-regulated, and 29 genes were down-regulated (Figure 3A,B). In order to further confirm the regulation trend of the AFN on the differential genes, 26 differential genes were screened out in AFN 227.50 mg/kg group, model group, and control group by the Venn diagram (Figure 3B), indicating that the AFN may be based on these genes to achieve the hypolipidemic effect.

**Figure 4:**
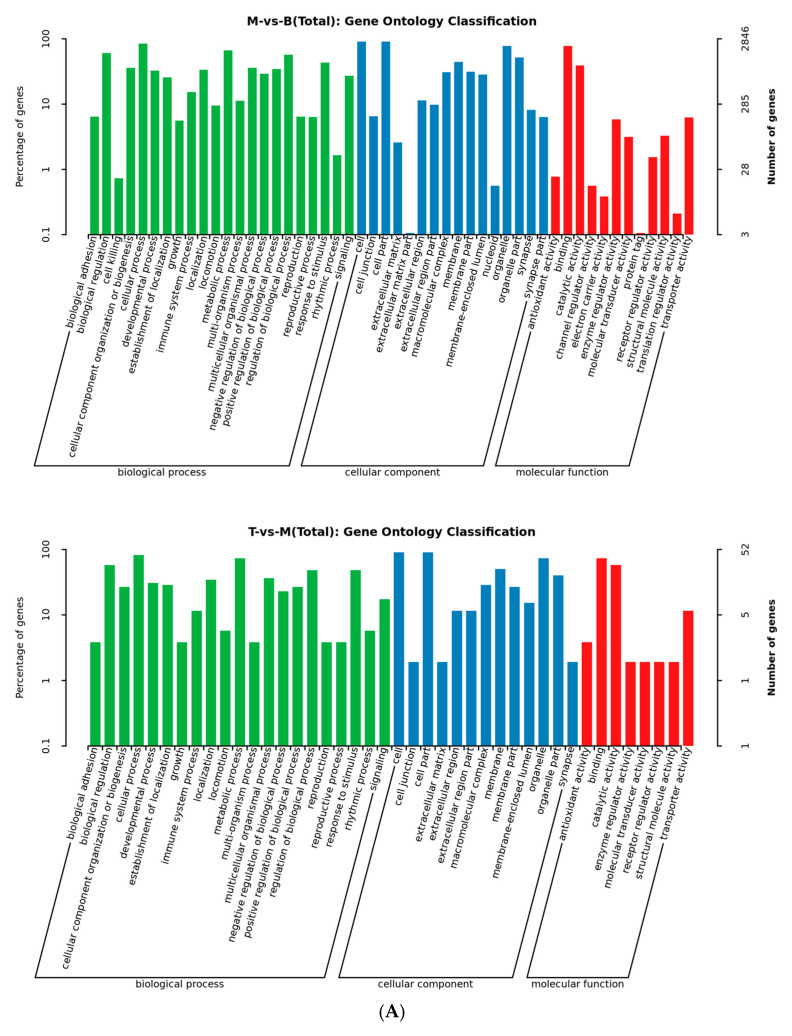

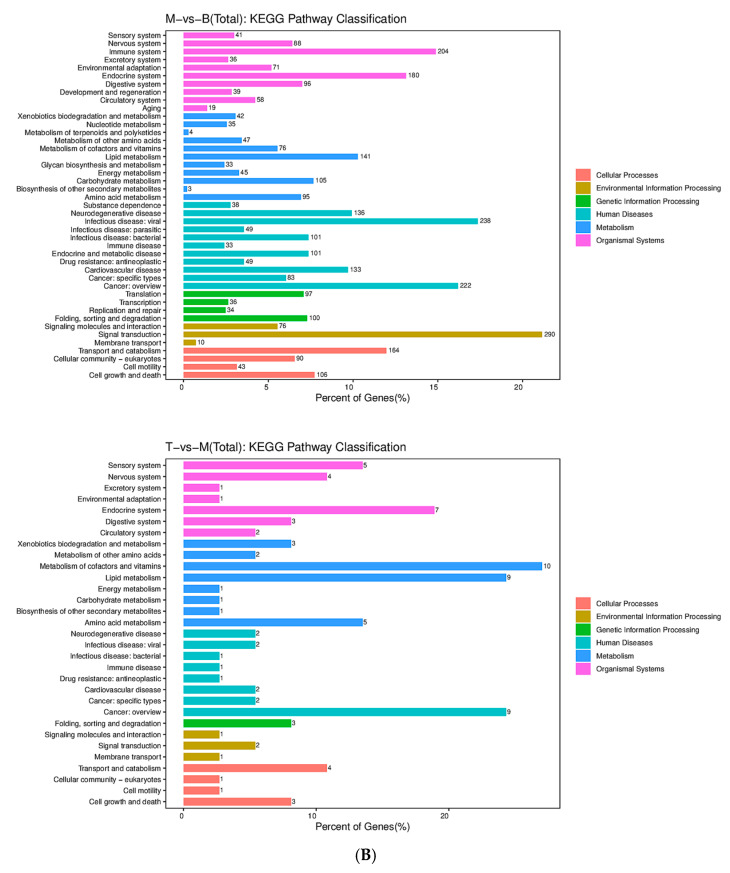
(**A**). GO functional enrichment analysis plot of control vs. model, AFN 341.25 mg/kg group vs. model. (**B**). KEGG pathway enrichment analysis plot of control vs. model, AFN 341.25 mg/kg group vs. model.

In order to further investigate the mechanism of the hypolipidemic effect of AFN, the GO function enrichment was performed on DEGs, and the results were categorized and analyzed in terms of molecular function (MF), cellular component (CC), and biological process (BP) for the targets (Figure 4A). The results suggest that AFN may exert hypolipidemic effects by affecting the activities of multiple key enzymes and then regulating related lipid metabolic processes. The KEGG pathways were significantly enriched for DEGs, and more than 10 pathways associated with hypolipidemic effects were identified in model group and AFN 341.25 mg/kg group (Figure 4B). The common pathways involved in the hypolipidemic effects in both groups included the PPAR signaling pathway (Figure S2), fat digestion and absorption pathway (Figure S3), and fatty acid degradation pathway (Figure S4).

### 2.4. Liquid-Mass Spectrometry Technique to Characterize the Components Absorbed into the Plasma at the AFN

**Figure 5:**
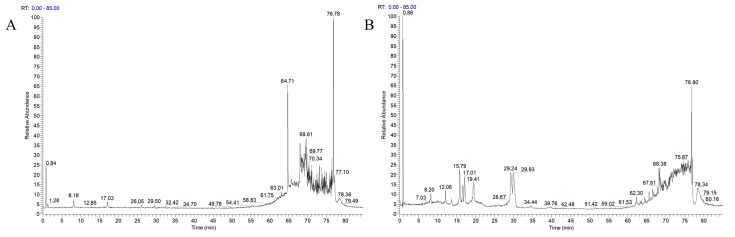
The total ion chromatographs (TIC) of UPLC-LTQ-Orbitrap-MS. AFN extraction in the positive ion mode (**A**) and negative ion mode (**B**).

Blank plasma and drug-containing plasma samples were analyzed using UPLC-MS^n^ to determine the active chemical components. The total ion flow diagrams of the samples in positive and negative ion modes characterized the AFN plasma absorbed ingredients (Figure 5). They were combined with the group’s previous analysis of AFN in vitro components [4] and relevant references, as well as based on secondary fragmentation information, a total of 30 chemical components were identified (Table 1). The prototypical forms of the 30 components include 2-hydroxy-1-methoxyaporphine, armepavine, *N*-norarmepavine, higenamine, nuciferine, *N*-nornuciferine, coclaurine, *N*-methylcoclaurine, *N*-methylisococlaurine, anonaine, lysicamine, caaverine and nuciferoline. The selection of these 13 alkaloidal prototype components from *N. folium* for subsequent research on the mechanism of hypolipidemic action was conducted both to elucidate the material basis and mechanistic pathways underlying the lipid-lowering effects of its alkaloids, and to better align with quality standard studies.

### 2.5. Ingredient-Target Molecular Docking

After topological analysis, the plasma-absorbed ingredients were ranked (Table S6), and the three ingredients of *N. folium*: nuciferine, *N*-nornuciferine, and *N*-methylisococlaurine were identified as the key ingredients of *N. folium* for the treatment of hyperlipidemic components. *CD36*, *FABP1*, *ACAA2*, *ACADVL*, *APOC3*, *APOA4*, *SLC27A4*, *PPARA*, and *CPT1A*, which were screened from the PPAR signaling pathway, the fat digestion and absorption pathway, and the fatty acid degradation pathway, were selected as docking target proteins for molecular docking (Table S7). In this study section, compounds with docking scores less than −5 and binding free energy scoring less than −30 kcal/mol were found to bind stably to the target proteins with some potential activity.

**Figure 6:**
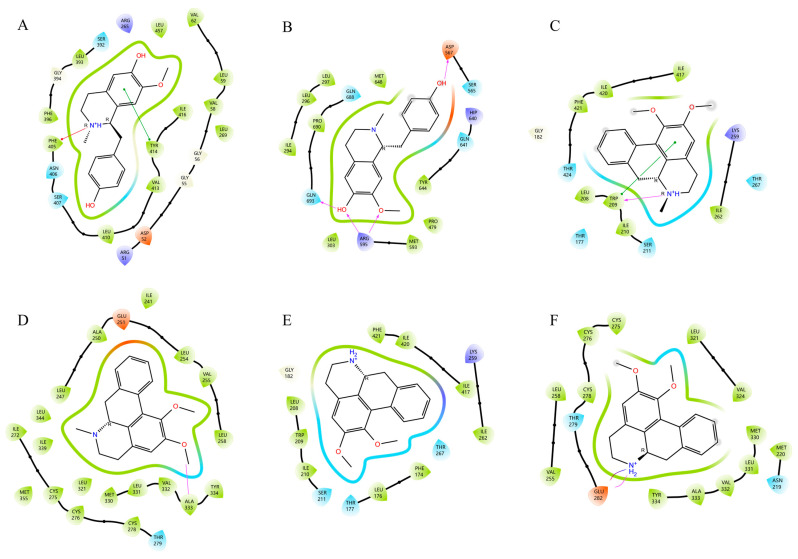
Two-dimensional molecular docking diagram of key active ingredients and proteins. (**A**) Molecular docking diagram of *N*-Methylisococlaurine with SLC27A4. (**B**) Molecular docking diagram of *N*-Methylisococlaurine with CPT1A. (**C**) Molecular docking diagram of Nuciferine with ACADVL. (**D**) Molecular docking diagram of Nuciferine with PPARA. (**E**) Molecular docking diagram of *N*-nornuciferine with ACADVL. (**F**) Molecular docking diagram of *N*-nornuciferine with PPARA.

According to the results of the XP Gscore score and MM-GBSA score, *N*-methylisococlaurine docked by forming a π-π bond with residue TYR414 of *SLC27A4* and a Pi-Cation bond with PHE405 (Figure 6A). *N*-methylisococlaurine docked by forming one hydrogen bond with residue ASP567 and GLN693 of *CPT1A*, respectively, and two hydrogen bonds with ARG595 (Figure 6B).

Nuciferine is docked by forming one hydrogen bond and one π-π bond with residue TRP209 of *ACADVL* (Figure 6C). Similarly, it is docked by forming a hydrogen bond with residue ALA333 of *PPARA* (Figure 6D).

The binding of *N*-nornuciferine to *ACADVL* mainly depends on hydrophobic interaction (Figure 6E). *N*-nornuciferine forms a hydrogen bond and a salt bridge with the GLU282 residue of *PPARA* (Figure 6F).

### 2.6. Verification by RT-qPCR

**Figure 7:**
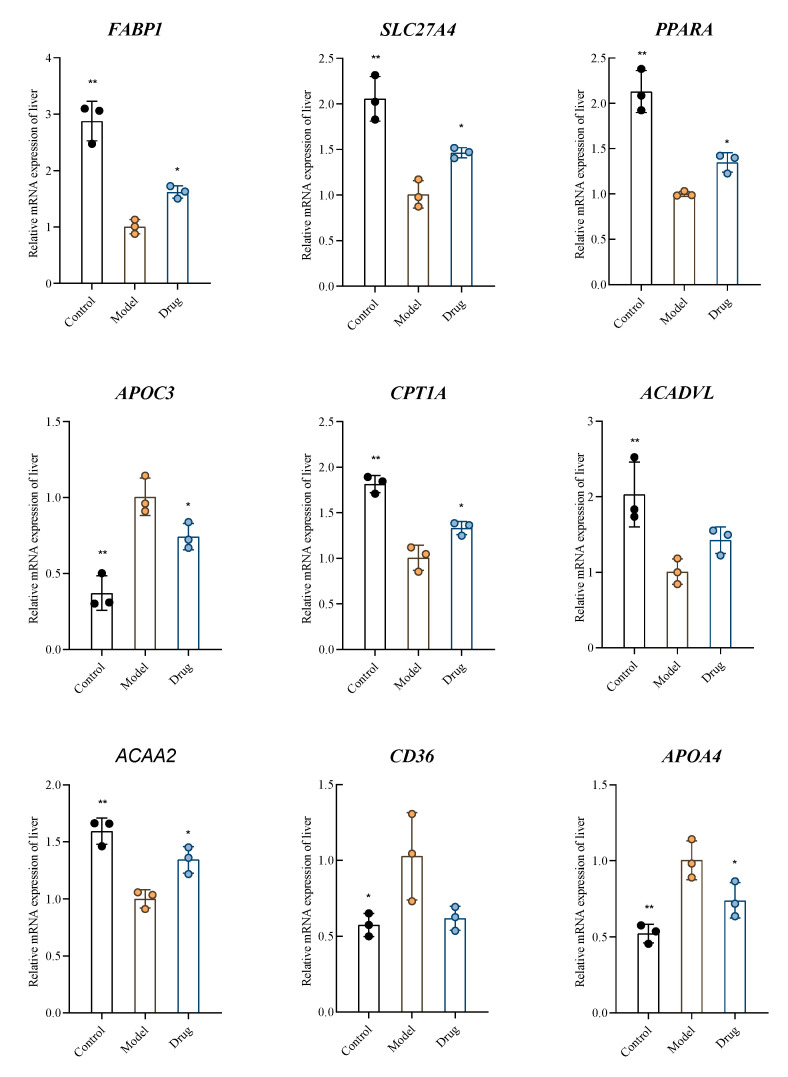
The results of key gene expression detected by RT-qPCR. All data are shown as the Mean ± SD (*n* = 6). * *p* < 0.05, ** *p* < 0.01 vs. model group.

The expression of 9 key genes, which were screened in transcriptome sequencing and could enhance the expression of hyperlipidemia-related pathways, was validated (Figure 7). The results showed that the expression of *FABP1*, *SLC27A4*, *PPARA*, *CPT1A*, *ACADVL*, and *ACAA2* mRNAs was significantly downregulated (*p* < 0.05). The expression of *APOC3*, *CD36*, and *APOA4* mRNAs was significantly up-regulated (*p* < 0.05) in the model control group compared to the blank control group after induction by the high-fat diet. The expressions of *FABP1*, *SLC27A4*, *PPARA*, *CPT1A*, and *ACAA2* mRNAs were significantly up-regulated, and *APOC3* and *APOA4* mRNA expressions were significantly down-regulated after AFN intervention compared with the model control group. The above results suggest that the AFN is involved in lipid metabolism by affecting the expression of *FABP1*, *SLC27A4*, *PPARA*, *CPT1A*, *ACAA2*, *APOC3*, and *APOA4* mRNAs, thus exerting a hypolipidemic effect.

## 3. Discussion

As a hypolipidemic drug, the research on the hypolipidemic mechanism of action of the AFN is relatively weak. We used a comprehensive strategy including plasma medicinal chemistry, transcriptome sequencing and molecular docking to clarify the hypolipidemic mechanism of the AFN by bi-directional screening of the components and the pathways of action.

According to the results of Cytoscape web analysis, Degree’s top-ranked compounds are nuciferine, *N*-nornuciferine, and *N*-methylisococlaurine. Nuciferine has hypolipidemic, lipid metabolism-regulating, anti-inflammatory, antioxidant, anti-atherosclerotic, and glucose metabolism-regulating effects [9] and can inhibit the expression of inflammatory factor genes, such as *NF-κB*, *IL-6*, and *TNF-α* from exerting an anti-inflammatory effect, and can reduce the formation of ox-LDL by lowering or inhibiting the formation of ox-LDL, decreasing the accumulation of lipids, promoting the excretion of bile acids, inhibiting the uptake of exogenous lipid, and alleviating the insulin *N*-nornuciferine can significantly inhibit the activities of α-glucoside, pancreatic lipase, and COX-2, and at the same time can inhibit lipid peroxidation and scavenge free radicals [10,11]. *N*-Methylisococlaurine belongs to the benzylisoquinoline alkaloid, and the same type of alkaloidal constituents were reported to have hypolipidemic effects in the literature [4], so it is inferred that it may also have hypolipidemic effects. These studies have demonstrated that nuciferine, *N*-nornuciferine, and *N*-methylisococlaurine possess hypolipidemic effects. The findings of our research further elaborate on the mechanisms of action of these three alkaloids, thereby laying a foundation for studies on the hypolipidemic effects of *N. folium*.

Through pathway enrichment of differential genes, three pathways, PPAR signaling pathway, fat digestion and absorption pathway, and fatty acid degradation pathway, were the focus of analysis. PPAR signaling pathway mediates a series of physiological activities such as fatty acid oxidation, cholesterol synthesis and catabolism, and plays a vital role in maintaining the homeostasis of lipid metabolism in the body, and the signaling pathway (Figure S2). According to the results of transcriptome sequencing, the differentially expressed genes involved in this signaling pathway included *CPT1A*, *CD36*, *SLC27A4*, *APOC3*, *FABP1*, and AFN was able to affect the expression of *CPT1A*, *ACADVL*, and *ACAA2* genes in the fatty acid degradation pathway after intervention in the hyperlipidemia model mice, and the fatty acid degradation pathway (Figure S3). The fat digestion and absorption pathway (Figure S4). AFN can affect the expression of *APOA4*, *FABP1*, *CD36* and *SLC27A4* genes in the fat digestion and absorption pathway.

The molecular docking technique is a method to predict the binding mode and affinity of proteins and small molecules by simulating and calculating their interactions to carry out the prediction of pharmacodynamic targets and drug screening [12,13], and it has been widely used in the screening of active ingredients as well as the study of the mechanism of action of traditional Chinese medicine (TCM). However, the molecular docking technique and MM-GBSA method are based on electronic computers to complete the experiments and make virtual predictions based on the binding modes and affinities. The good or lousy docking results do not all represent accurate results, so RT-qPCR subsequently validated the present study to ensure the reliability of the experiments.

The above 9 targets, including 4 targets that successfully docked with 3 alkaloid molecules, were verified by PCR, and the mechanism of the 7 targets that were in line with the trend (including the above four targets) exerted their hypolipidemic function was studied as follows.

Among them, *FABP1* is mainly involved in regulating the uptake and metabolism of fatty acids and other lipid molecules, and it can promote cellular uptake of fatty acids by elevating the intracellular fatty acid concentration gradient, which minimizes the amount of unbound fatty acids in the cell to the greatest extent possible [14].

Adipocyte insulin resistance promotes the hydrolysis of fatty triglycerides with concomitant elevation of plasma and hepatic free fatty acid levels, and genetic studies in humans and mice have demonstrated that mutations and polymorphisms in the *SLC27A4* gene are associated with insulin resistance and obesity [15].

*PPARA* is an essential regulator of lipid metabolism in liver tissue, which can effectively activate multiple lipid metabolism pathways in liver tissue. It can bind to specific ligands to form a ligand-activated complex and then induce protein expression by activating target genes, thus regulating lipid metabolic pathways such as fatty acid uptake, oxidation, synthesis, lipid transport, and lipoprotein assembly, and finally realizing the homeostasis of energy metabolism and lipids [16].

The *CPT1A* gene also plays an essential role in fatty acid metabolism. It is also responsible for transporting long-chain key rate-limiting enzymes to transport fatty acids from the cytosol to the mitochondria for the β-oxidation energy supply. Tingting Luo et al. [17] used palmitic acid to induce a high-fat model in HepG2 cells. They found that methylxanthines improved cellular lipid deposition by up-regulating *CPT1A* gene expression levels. In the PPAR signaling pathway, the AFN may activate the expression of *CPT1A* by up-regulating *CPT1A* gene expression, which in turn promotes β-oxidation of fatty acids to provide energy for the body and reduces fatty acid accumulation as well as lipid deposition in the body, thus realizing the effect of lowering blood lipids [13,18].

*ACAA2* is mainly involved in the β-oxidation of fatty acids, anabolic metabolism of cholesterol, and metabolism of bile acid. De Boer et al. [19] found that quercetin altered fatty acid metabolism in rats by up-regulating the expression levels of *ACAA2*, *ECH1*, and *ACOX1* and led to a decrease in plasma fatty acid levels.

*APOC3* is a key inhibitor of LPL, predominantly found in TRL. LPL influences TRL metabolism because LPL is the rate-limiting enzyme for the reaction of TG catabolism to glycerol and free fatty acids [20,21].

Furthermore, under high-fat chow-feeding conditions, *APOA4* knockout mice have lower body weight and adipose tissue mass than C57BL6 mice, while distal gut hormones are more responsive to a high-fat diet [22]. It was shown that *APOA4* overexpressing mice had significantly increased hepatic TG secretion rate and *VLDL* secretion [23]. In vivo research experiments demonstrate that plasma *APOA4* can affect plasma TC levels by promoting the process of reverse cholesterol transport [24,25].

The integrated strategy of plasma medicinal chemistry, transcriptome sequencing and molecular docking was used for the first time in the study of the hypolipidemic effects of AFN. We screened out the material basis of action in the blood-entry components in the organism from the changes in chemical components after the interaction of body and drug, while transcriptome sequencing was used to study the overall pathway of hypolipidemic effects from the changes in the body, then linking results from both sides together by molecular docking to reveal the interaction of the body and the alkaloid fraction, finally, the mechanism of the alkaloid fraction for lipid-lowering was clarified. This innovative approach provides a new solution way to the problem of multiple components and targets in hypolipidemic of *N. folium*.

## 4. Materials and Methods

### 4.1. Preparation of AFN

A total of 3.7 kg *of N. folium* was purchased from Beijing Tong Ren Tang Technology Development Co., Ltd., Beijing, China. and was extracted three times with 16 times the amount of 90% ethanol solvent, each for 1.5 h. After the filtrate was concentrated, it was ultrasonically dispersed with ten times the amount of 1% HCl and then centrifuged. The supernatant was passed through D001-CC macroporous cation exchange resin. After adsorption, 5 BV 50% ethanol was used to remove impurities. Then, 10 BV of 1% ammoniacal ethanol was used to elute the alkaloid components, which were collected. The solvent was then recovered under reduced pressure, and the residue was dried to obtain AFN 55.52 g (yield 1.50%).

### 4.2. Determination of AFN by High-Performance Liquid Chromatography

Ten batches of *N. folium* medicinal materials (S1–S10) were purchased from pharmacies and medicinal materials markets nationwide to prepare ten batches of AFN. Armepavine reference substance (batch number: P22M10F88910), *N*-nornuciferine reference substance (batch number: W29N7Z25888), and nuciferine reference substance (batch number: W17N8Z48436) were purchased from Shanghai Yuanye Bio-Technology Co., Ltd., Shanghai, China; the reference substance of 2-hydroxy-1-methoxyaporphine (Batch No.: DST200511-146) was purchased from Chengdu DeSiTe Biological Technology Co., Ltd., Chengdu, China. Took ten batches of AFN 20 mg each, accurately weighed, dissolved in methanol, and diluted in a 50 mL volumetric flask. All solutions were filtered through a 0.45 μm membrane. Samples were transferred to injection bottles for HPLC analysis (Tables S1 and S2).

### 4.3. Hyperlipidemic Mouse Model

The animal experiment was approved by the Animal Ethics Committees of Beijing University of Chinese Medicine (Registration No. BUCM-2022100903-4092). The date of ethical approval is 9.3, 2021. Male C57BL/6 mice aged 6–8 weeks weighing about 18 g were purchased from SPF (Beijing) BIOTECHNOLOGY Co., Ltd., Beijing, China. The license number for producing experimental animals is SCXK (Jing) 2019-0010. All mice were housed at 22–24 °C in a barrier-free environment, with a relative humidity of 50–70% and a 12/12 h light/dark cycle. After one week of acclimatization, the mice were randomly assigned to 6 groups: control group, hyperlipidemia model group, AFN 113.75 mg/kg group, AFN 227.50 mg/kg group, AFN 341.25 mg/kg group and simvastatin 15.17 mg/kg group. The mice, except the control group, were fed with a high-fat diet for four weeks to establish a hyperlipidemia model. At the same time, the mice in the control group were fed a normal diet. The mice in medicinal treated groups were administered relative solvent by intragastric administration. After the last administration, mice were fasted for 12 h. Then, the serum and liver samples were collected and stored at −80 °C.

### 4.4. Determination of Serum and Liver Biochemical Indices

According to the kit instructions (Beckman Coulter Laboratory Systems Co., Ltd., Suzhou, China), the contents of TC, TG, HDL-C, LDL-C, AST and ALT in serum were determined by the automatic biochemical analyzer (Beckman Coulter Commercial ENTERPRISE (China) Co., Ltd., Shanghai, China). Approximately 0.1 g of liver tissue was collected from each mouse, and 9 volumes of PBS buffer were added. The mixture was thoroughly homogenized using a homogenizer under ice-water bath conditions to prepare a 10% mouse liver homogenate. After low-temperature centrifugation (3000 r/min, 20 min), the supernatant was aspirated and reserved for subsequent use. In accordance with the kit instructions, the contents of TC and TG in mouse liver tissues were determined using an automatic biochemical analyzer. All data are expressed as mean ± standard deviation ($\bar{x}$ *± s*). Statistical analysis was performed using SPSS 23.0 software. Comparisons between two experimental groups were analyzed by t-test, and comparisons among multiple experimental groups were analyzed by one-way analysis of variance (ANOVA). *p* < 0.05 indicates a significant difference, *p* < 0.01 indicates a highly significant difference.

### 4.5. Transcriptomics by RNA-Sequencing

Total RNA was extracted from 100 mg of liver samples from the control group (B), model group (M), and the AFN 341.25 mg/kg group (T) using TRlzol reagent (Invitrogen Trading Co., Ltd., Shanghai, China) according to the instructions. The library was quantified by Agilent 2100 Bioanalyzer (Agilent Technologies (China) Co., Ltd., Beijing, China). Then, it was sequenced on a NovaSeq 6000 platform (Illumina) by Shanghai Ouyi Biomedical Technology Co., Ltd., Shanghai, China. The number of counts of each sample gene was normalized using DESeq2 [26] software (Base Mean values were used to estimate expression), and differential folds were calculated; DEGs were defined as genes with | log2 (FC) | > 1 and a significance *p*-value < 0.05. DEGs were analyzed for GO and KEGG enrichment. The Benjamini–Hochberg (BH) method was used to adjust the *p*-values. A GO term was considered significantly enriched when the adjusted *p*-value < 0.05. The significance of KEGG enrichment for differential genes in each Pathway entry was calculated using the hypergeometric distribution test method.

### 4.6. UPLC-MS^n^ Analysis of AFN Plasma-Absorbed Ingredients

This study used two groups of male Wistar rats (weighing about 170 g). They were all purchased from the SPF (Beijing) BIOTECHNOLOGY Co., Ltd. Breeding conditions were the same as the efficacy experiments in this study.

In the first group, four rats were intragastric administrated with 1 mL/100 g of purified water, and 1 h later, blood was collected from the retroorbital venous plexus and centrifuged (4000 r/min, 10 min, 4 °C). It took 300 µL of plasma, 5 times the amount of methanol was added, vortexed for 3 min, centrifuged again (13,000 r/min, 10 min), and the supernatant was concentrated to dryness. The residue was reconstituted with 150 µL of 30% methanol, and the supernatant was centrifuged (140,000 r/min, 15 min) and used as a blank plasma sample.

In the second group, four rats were also taken and administered with the AFN concentration of 132.3 mg/kg, and the samples were prepared according to the same method as the blank control group and were used as drug-containing plasma samples for reserve.

The drug-containing serum was transferred to sample vials, and the samples were subjected to UPLC-MS^n^ analysis. The analytical methods are provided in Tables S3 and S4.

The compounds represented by those peaks in the mass spectrum were identified by Xcalibur and Compound Discoverer (version 3.0).

### 4.7. Computational Analysis

Cytoscape (3.9.0 software) was used to screen plasma-absorbed components for key components with “degree, DC”, “mediated centrality, BC” and “near centrality, CC”. The three components screened were nuciferine, *N*-nornuciferine, and *N*-methylisococlaurine. Crystal structures corresponding to the target proteins *CD36*, *FABP1*, *ACAA2*, *ACADVL*, *APOC3*, and *APOA4* proteins were obtained from the RCSB PDB database. AlphaFold predicted the protein crystal structures of *SLC27A4*, *PPARA*, and *CPT1A*. The Protein Preparation Wizard module of Schrödinger software was used for their processing. The 3D structures of nuciferine, *N*-nornuciferine, and *N*-methylisococlaurine in SDF format were obtained by PubChem. Schrödinger software was used to obtain the active sites of the proteins. The processed ligands of the three compounds were molecularly docked to the active sites of each of the nine proteins (using the highest precision XP docking), and ten conformations were taken from each molecule for flexible docking, and their average results were generated. The binding energy calculations of the three compound ligands were analyzed by the MM-GBSA method with the active sites of nine proteins, respectively. Compounds with a XP GScore less than −5 and a MM-GBSA dG Bind less than −30 kcal/mol bind stably to target proteins, exhibit certain potential activity, and are considered successfully docked.

### 4.8. Real-Time PCR

Combining transcriptome sequencing results, 9 potential genes were screened out. The primer sequences are presented in Table 2. The PerfectStart^TM^ Green qPCR SuperMix kit was employed to assess mRNA expression. Each 10 µL reaction mixture contained 5 µL of 2× PerfectStart™ Green qPCR SuperMix, 0.2 µL of each forward and reverse primer (10 µM), 2 µL of the diluted cDNA template, and 2.6 µL of nuclease-free water. The amplification protocol consisted of an initial denaturation at 94 °C for 30 s, followed by 45 cycles of denaturation at 94 °C for 5 s, and a combined annealing/extension step at 60 °C for 30 s. After the amplification cycles, a melting curve analysis was performed by gradually increasing the temperature from 60 °C to 97 °C with 5 fluorescence acquisitions per °C to confirm the specificity of the amplification products. Relative transcript levels were evaluated using the 2^−ΔΔCT^ method.

## 5. Conclusions

The results of this study verified the hypolipidemic efficacy of AFN. RNA-seq was combined to demonstrate that targets in three pathways (the PPAR signaling pathway, the fat digestion and absorption pathway, and the fatty acid degradation pathway) are regulated by ANF. Meanwhile, based on the analysis of plasma components combined with molecular docking technology, the hypolipidemic mechanism of three specific components of AFN, nuciferine, *N*-nornuciferine, and *N*-methylisococlaurine, in combination with the relevant targets, was hypothesized and preliminarily verified by PCR. The preliminary validation was carried out by PCR, which lays the foundation for further in-depth and systematic research on the material basis and mechanism of the hypolipidemic effect of *N. folium*.

## Figures and Tables

**Table 1:** Results of chemical characterization of plasma absorption of alkaloid ingredients in AFN.

No.	RT (Min)	Chemical Formula	Theoretical *m*/*z*	Measured (*m*/*z*)	Mass Error (ppm)	MS/MS (*m*/*z*)	Compound
1	6.01	C_22_H_26_NO_9_	448.16020	448.15952	−1.535	272.12759, 255.10112, 237.09047, 161.05951, 107.04939	Norcoclaurine-Glucuronide
2	7.71	C_24_H_30_NO_9_	476.19150	476.19080	−1.487	300.15881, 269.11661, 237.09048, 175.07512, 107.04935	*N*-Methylcoclaurine-Glucuronide
3	8.08	C_23_H_28_NO_9_	462.17585	462.17502	−1.813	286.14325, 269.11673, 175.07516, 107.04937	*N*-Norarmepavine desmethyl-Glucuronide
4	8.20	C_23_H_28_NO_9_	462.17585	462.17499	−1.878	286.14322, 269.11667, 237.09055, 209.09575, 175.07513, 107.04935	Coclaurine-Glucuronide
5	8.73	C_24_H_30_NO_9_	476.19150	476.19092	−1.235	300.15894, 283.13239, 252.11403, 189.09076, 107.04939	Armepavine desmethyl-Glucuronide
6	9.54	C_23_H_26_NO_8_	444.16529	444.16467	−1.403	268.13269, 251.10629, 236.10664, 219.08018, 191.08528	Caaverine-Glucuronide
7	9.86	C_24_H_30_NO_9_	476.19150	476.19095	−1.172	300.15894, 283.13245, 252.11414, 189.09076, 107.04940	Armepavine desmethyl-Glucuronide
8	11.04	C_25_H_32_NO_9_	490.20715	490.20657	−1.199	314.17447, 283.13239, 252.11391, 189.09074, 107.04939	Armepavine Glucuronide
9	11.33	C_24_H_30_NO_9_	476.19150	476.19089	−1.298	300.15881, 283.13232, 251.10623, 175.07518, 107.04935	*N*-Methylisococlaurine-Glucuronide
10	11.67	C_23_H_26_NO_8_	444.15783	444.15726	−1.374	268.13269, 237.09061, 219.08018, 191.08527	*N*-Nornuciferine desmethyl-Glucuronide
11	11.69	C_23_H_28_NO_9_	462.17585	462.17499	−1.878	286.14319, 269.11664, 175.07510, 107.04935	*N*-Norarmepavine desmethyl-Glucuronide
12	11.81	C_23_H_28_NO_9_	462.17585	462.17508	−1.683	286.14328, 269.11670, 237.09074, 178.08604, 107.04937, 143.04901	Coclaurine-Glucuronide
13	12.05	C_24_H_30_NO_9_	476.19150	476.19098	−1.109	300.15894, 283.13245, 252.11407, 189.09079, 107.04939	Armepavine desmethyl-Glucuronide
14	12.50	C_23_H_26_NO_8_	444.16529	444.16467	−1.403	268.13269, 251.10628, 236.08281, 219.08020, 191.08527	Caaverine-Glucuronide
15	13.19	C_23_H_28_NO_9_	462.17585	462.17529	−1.229	286.14334, 269.11679, 237.09071, 209.09579, 178.08606, 143.04903, 107.04940	Coclaurine-Glucuronide
16	13.25	C_23_H_26_NO_8_	444.17846	444.17834	−1.538	268.13263, 251.10620, 219.08014, 191.08524	*N*-Nornuciferine desmethyl-Glucuronide
17	13.47	C_24_H_28_NO_8_	458.18094	458.18027	−1.469	282.14844, 251.10628, 219.08020, 191.08528	*O*-Nornuciferine-Glucuronide
18	16.60	C_17_H_18_NO_5_S	348.09001	348.08914	−0.579	268.13257, 251.10611, 236.08261, 219.08008, 191.08517	Caaverine-Sulfate
19	17.73	C_18_H_20_NO_5_S	362.10566	362.10507	−1.374	282.14847, 251.10629, 219.08022, 191.08017	*O*-Nornuciferine-Sulfate
20	17.82	C_18_H_20_NO_2_	282.14885	282.14841	−1.579	265.12198, 251.10623, 236.08275, 219.08017, 191.08527	*O*-Nornuciferine *
21	17.85	C_17_H_18_NO_5_S	362.09001	362.10495	−1.143	282.14838, 265.28619, 250.12221	*O*-Nornuciferine-Sulfate
22	21.26	C_17_H_12_NO_3_	278.08116	278.08063	−1.941	263.05716, 250.08626, 235.06229	Desmethyl lysicamine
23	21.68	C_17_H_18_NO_2_	268.13320	268.13266	−2.034	251.10620, 236.08276, 219.08009, 191.08519	Caaverine
24	25.73	C_18_H_18_NO_2_	280.13320	280.13275	−1.625	26310638, 248.08281, 233.05934, 217.78973, 203.88098	Dehydro-*N*-Nornuciferine-Glucuronide
25	26.30	C_24_H_28_NO_8_	458.18094	458.18008	−1.884	282.14838, 251.10623	*N*-Nornuciferine-Glucuronide
26	29.59	C_17_H_16_NO_2_	266.11755	266.12531	−2.124	249.09058, 235.10686, 219.08014	Anonaine
27	29.79	C_18_H_20_NO_2_	282.14885	282.14835	−1.791	265.12192, 250.09842, 234.10358	*N*-Nornuciferine *
28	30.35	C_19_H_22_NO_2_	296.16450	296.16400	−1.707	265.12189, 250.09836, 234.10355	Nuciferine *
29	34.58	C_18_H_14_NO_3_	292.09681	292.09631	−1.745	277.07294, 248.07016	Lysicamine
30	34.95	C_19_H_22_NO_3_	312.15942	312.15869	−2.339	265.12183, 250.09834, 234.10350	Nuciferoline

Note: “*” indicates that a comparison has been made with a standard.

**Table 2:** Primer sequences.

Primer Name	Forward Primer (5′–3′)	Reverse Primer (5′–3′)
*FABP1*	GTGGTCCGCAATGAGTTC	CACCTTCCAGCTTGACGA
*SLC27A4*	GATTCTCCCTGTTGCTCCTGTA	TATCTCTCCTGACCGTCTTGAT
*PPARA*	GACTCTAAAGATCAGATTCCGC	GTTGAGCTGGTCTAGATCGCA
*APOC3*	AGAAGGCTTGGGACTCAT	CTCTACCTCTTCAGCTCGG
*CPT1A*	ACCTTGGACCCAAATTGC	ATGTATTCCTCCCACCAGTCA
*ACADVL*	TCTGCCCAGCGACTTTAT	TGGTGGAAGCATCAGAGGA
*ACAA2*	TCTGGTTTCCAGTCCATCG	TCTCTGTTCCTCCACACAAG
*CD36*	GTCCTTACACATACAGAGTTCG	CTCTGTTCCAACAGACAGTGA
*APOA4*	GGTGGAGCCAACTCAAGAA	GGCCTCTTGGACTTTAGTG

## Data Availability

The original contributions presented in this study are included in the article/Supplementary Material. Further inquiries can be directed to the corresponding authors.

## Supplementary Materials

The following supporting information can be downloaded at https://www.mdpi.com/article/10.3390/molecules30183727/s1, Table S1: The parameters of the HPLC; Table S2: Gradient elution procedure of HPLC; Table S3: The parameters of UPLC-MS^n^; Table S4: Gradient elution procedure of UPLC-MS^n^; Table S5: Results of the determination of alkaloids in 10 batches of AFN; Table S6: Values for network analysis of plasma-absorbed active ingredients in AFN; Table S7: Binding energy information of key active ingredients of AFN to target proteins; Figure S1: The stacked HPLC traces of 10 batches of *N. folium*; Figure S2: The PPAR signaling pathway; Figure S3: The fat digestion and absorption pathway; Figure S4: The fatty acid degradation pathway.

## Abbreviations

ACAA2: Acetyl-Coenzyme Acyltransferase; ACADVL, acyl-Coenzyme A dehydrogenase; alkaloid fraction of *Nelumbinis folium*, AFN; ALT, alanine transaminase; APOA4, Apolipoprotein A-IV; APOC3, apolipoprotein C3; AST, aspartate aminotransferase; CD36, Cluster of differentiation 36; CPT1A, Carnitine palmitoyltransferase; FABP1, fatty acid binding protein; GO, Gene Ontology; HDL-C, High-density lipoprotein cholesterol; HepG2, Human hepatocellular liver carcinoma cell line; KEGG, Kyoto Encyclopedia of Genes and Genomes; LDL-C, Low-density lipoprotein cholesterol; MM-GBSA, Molecular Mechanics Generalized Born Surface Area; PPARA, Peroxisome Proliferator Activated Receptor Alpha; RT-qPCR, Quantitative real-time PCR; SLC27A4, solute carrier family 27 member 4; TC, total cholesterol; TCM, Traditional Chinese Medicine; TG, triglyceride; UPLC-MS^n^, UPLC-LTQ-Orbitrap-MS.
