# Supplementary material for: Combination of Plasma Pharmacochemistry, RNA-Seq, and Molecular Docking Strategies to Reveal the Mechanism of the Alkaloid Fraction of Nelumbinis folium for the Treatment of Hyperlipidemia
Source: Molecules. 2025 Sep 12;30(18):3727. doi: 10.3390/molecules30183727 (PMC12472672; doi:10.3390/molecules30183727)
Supplement: Supplementary file 1 [file molecules-30-03727-s001.zip › molecules-3827768-supplementary.pdf]

Supplementary Information

Table S1 The parameters of the HPLC.

Parameters	Conditions
Workstation	Waters e2695 high-performance liquid chromatography system (four-component gradient pump, 2707 automatic sampler, 2998 PDA detector, Empower chromatographic workstation, USA)
Chromatographic column	Sunfire C18 column (4.6 mm × 250 mm, 5 μm)
Column temperature	30 °C
Flow rate	1.0 ml/min
Detection wavelength	270 nm and 285 nm
Injection volume	20 μL
Mobile phase	Acetonitrile (A) 0.1 % triethylamine - water (B)

Table S2 Gradient elution procedure of HPLC.

Time	Ratio
0-10min	25 % A
10 ~ 25min	25 % ~ 40 % A
25 ~ 30 min	40 % ~ 60 % A

Table S3 The parameters of UPLC-MS^n.

Parameters	Conditions
Workstation	Thermo ScientificTM LTQ Orbitrap XLTM (UPLC-MS) system
Chromatographic column	WATERS ACQUITY UPLCTM BEH C18 (50 mm × 2.1 mm, 1.7 μm)
Column temperature	35 ℃
Flow rate	0.3 mL/min
Detection wavelength	280 nm
Injection volume	5 μL
Mobile phase	0.2% formic acid in methanol (A) 0.2% formic acid in water (B)
ESI ion source in positive and negative ion modes	4.0 kV and - 3.5 kV
Ion source temperature	320 ℃
Scanning range of m/z	100~1200 Da

Table S4 Gradient elution procedure of UPLC-MS^n.

Time	Ratio
0 ~ 4 min	4% A
4 ~ 8 min	4 ~ 9% A
8 ~ 16 min	9 ~ 14.3% A
16 ~ 24 min	14.3 ~ 18% A
24 ~ 28 min	18 ~ 23.7% A
28 ~ 33 min	23.7 ~ 25.4% A
33 ~ 37 min	25.4 ~ 27.5% A
37 ~ 40.5 min	27.5 ~ 30.5% A
40.5 ~ 44.5 min	30.5 ~ 34.5% A
44.5 ~ 48.5 min	34.5 ~ 36.5% A
48.5 ~ 51.5 min	36.5 ~ 39.5% A
51.5 ~ 55 min	39.5 ~ 50% A
55 ~ 59 min	50 ~ 65% A
59 ~ 66 min	65 ~ 77% A
66 ~ 70 min	77 ~ 86% A
70 ~ 85 min	86 ~ 100% A

Table S5 Results of the determination of alkaloids in 10 batches of AFN.

Batch	Determination/%				
	Armepavine	2-Hydroxy-1-Methoxyaporphine	*N*-Nornuciferine	Nuciferine	Total determination
1	1.71124	3.61142	8.60229	20.87754	34.80249
2	0.64860	6.00911	11.96747	56.62330	75.24848
3	1.07774	3.48490	9.04564	36.13589	49.74417
4	1.89904	4.05000	11.27918	19.08460	36.31283
5	1.82151	2.22527	7.66069	19.83899	31.54646
6	1.35959	3.17679	8.16957	23.28969	35.99564
7	0.97436	3.95301	5.11529	22.80216	32.84482
8	1.20962	1.54603	4.21542	10.71108	17.68215
9	1.79456	3.79549	9.92794	21.04005	36.55804
10	1.40276	4.19782	5.79743	18.45525	29.85327

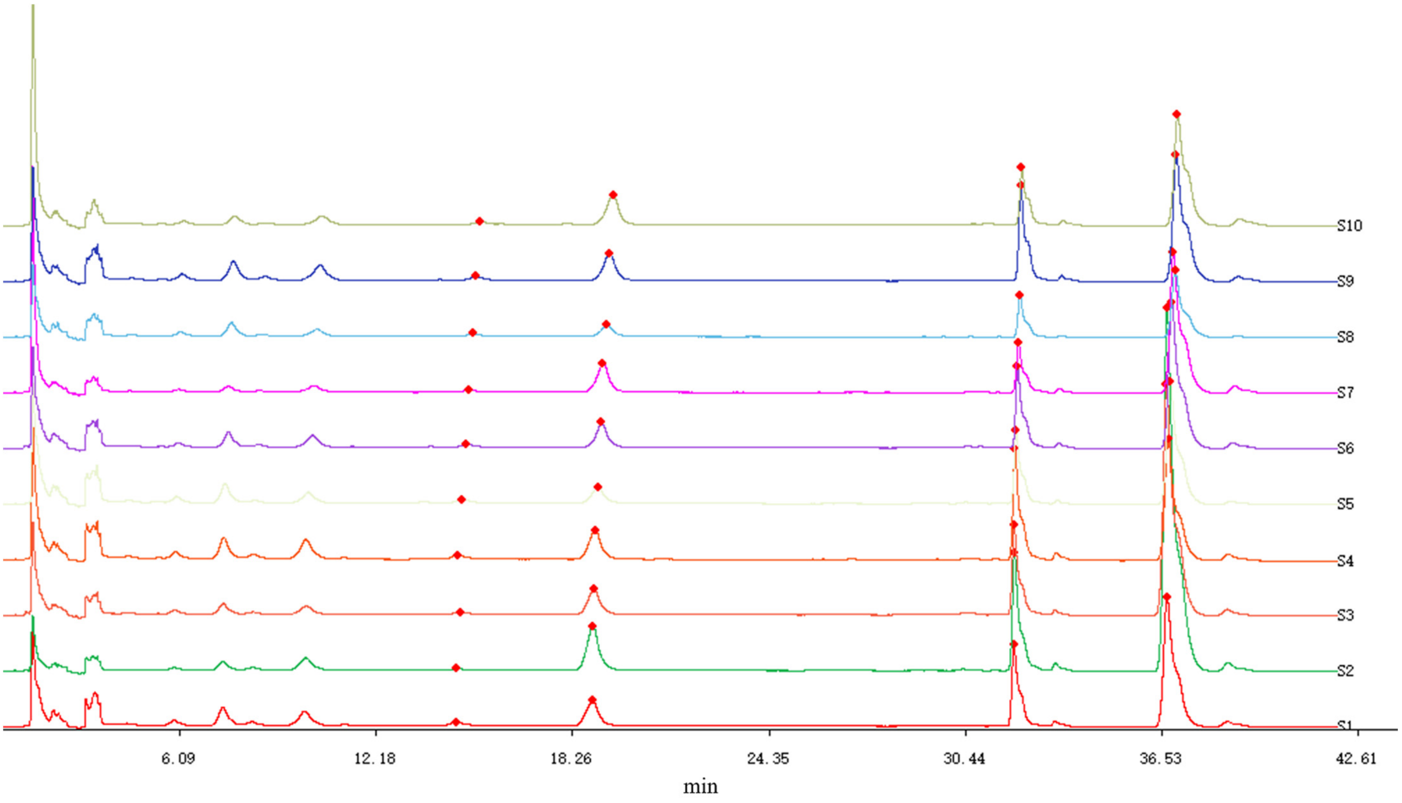

Figure S1 The stacked HPLC traces of 10 batches of *N. Folium.*

Table S6 Values for network analysis of plasma-absorbed active ingredients in AFN.

Active components	No.	Betweenness Centrality	Closeness Centrality	Degree
Nuciferine	E	0.25587488	0.545454545	22
N-Nornuciferine	F	0.098909157	0.521739130	20
N-Methylisococlaurine	I	0.096320084	0.510638298	19
Caaverine	L	0.051206052	0.510638298	19
Higenamine	D	0.054653418	0.500000000	18
Coclaurine	G	0.066992921	0.500000000	18
Lysicamine	K	0.102405871	0.500000000	18
N-Methylcoclaurine	H	0.025326255	0.489795918	17
Nuciferoline	M	0.081327096	0.489795918	17
N-Norarmepavine	C	0.030081976	0.480000000	16
Armepavine	B	0.012062991	0.461538462	14
2-Hydroxy-1-Methoxyaporphine	A	0.020014353	0.452830189	13
Anonaine	J	0.010853316	0.452830189	13

Table S7 Binding energy information of key active ingredients of AFN to target proteins.

Compound Name	Target protein	XP GScore	MM-GBSA dG Bind (kcal/mol)
Nuciferine （A）	FABP1	-1.237	-24.27
	SLC27A4	1.118	20.63
	PNLIP	-8.479	-47.15
	PPARA	-7.100	-34.69
	ACADVL	-5.883	-45.63
	CPT1A	-4.104	-25.25
	APOC3	-2.821	-23.38
	ACAA2	-4.299	-38.35
	APOA4	-4.006	-37.81
N-Nornuciferine （B）	FABP1	-1.261	-16.40
	SLC27A4	-1.724	-29.18
	PNLIP	-8.533	-49.55
	PPARA	-5.612	-44.94
	CPT1A	-4.315	-38.13
	APOC3	-2.218	-26.78
	ACADVL	-5.846	-46.66
	ACAA2	-4.092	-35.60
	APOA4	-3.960	-41.95
N-Methylisococlaurine （C）	FABP1	-7.620	-7.58
	SLC27A4	-5.308	-30.80
	PNLIP	-8.350	-43.80
	PPARA	-8.772	-19.86
	CPT1A	-6.332	-31.28
	ACADVL	-6.209	-48.20
	ACAA2	-4.840	-38.75
	CD36	-3.891	-23.40
	APOC3	-4.010	-15.14
	APOA4	-4.360	-40.21

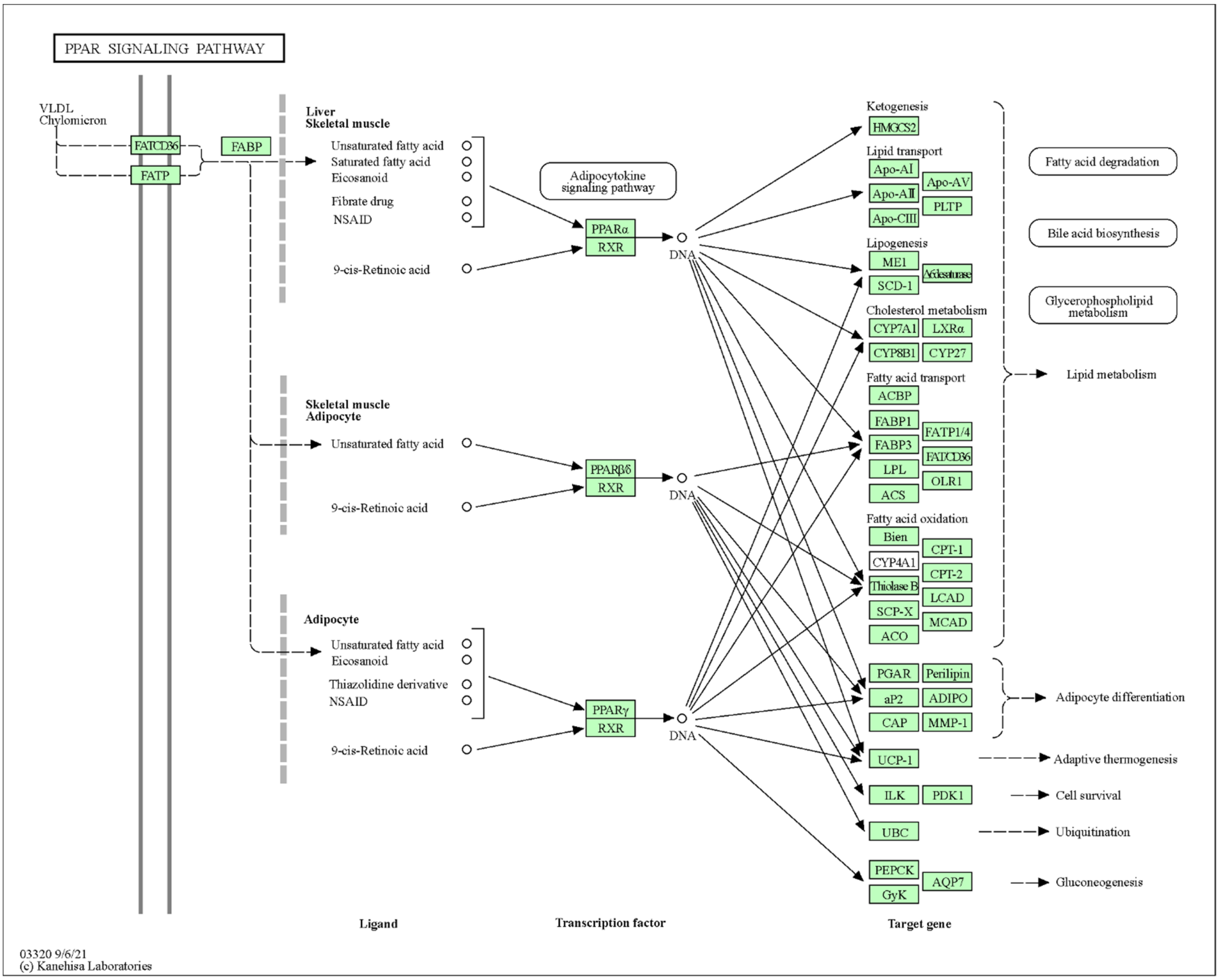

Figure S2 The PPAR signaling pathway. The figures are referenced from the KEGG PATHWAY Database (https://www.genome.jp/kegg/).

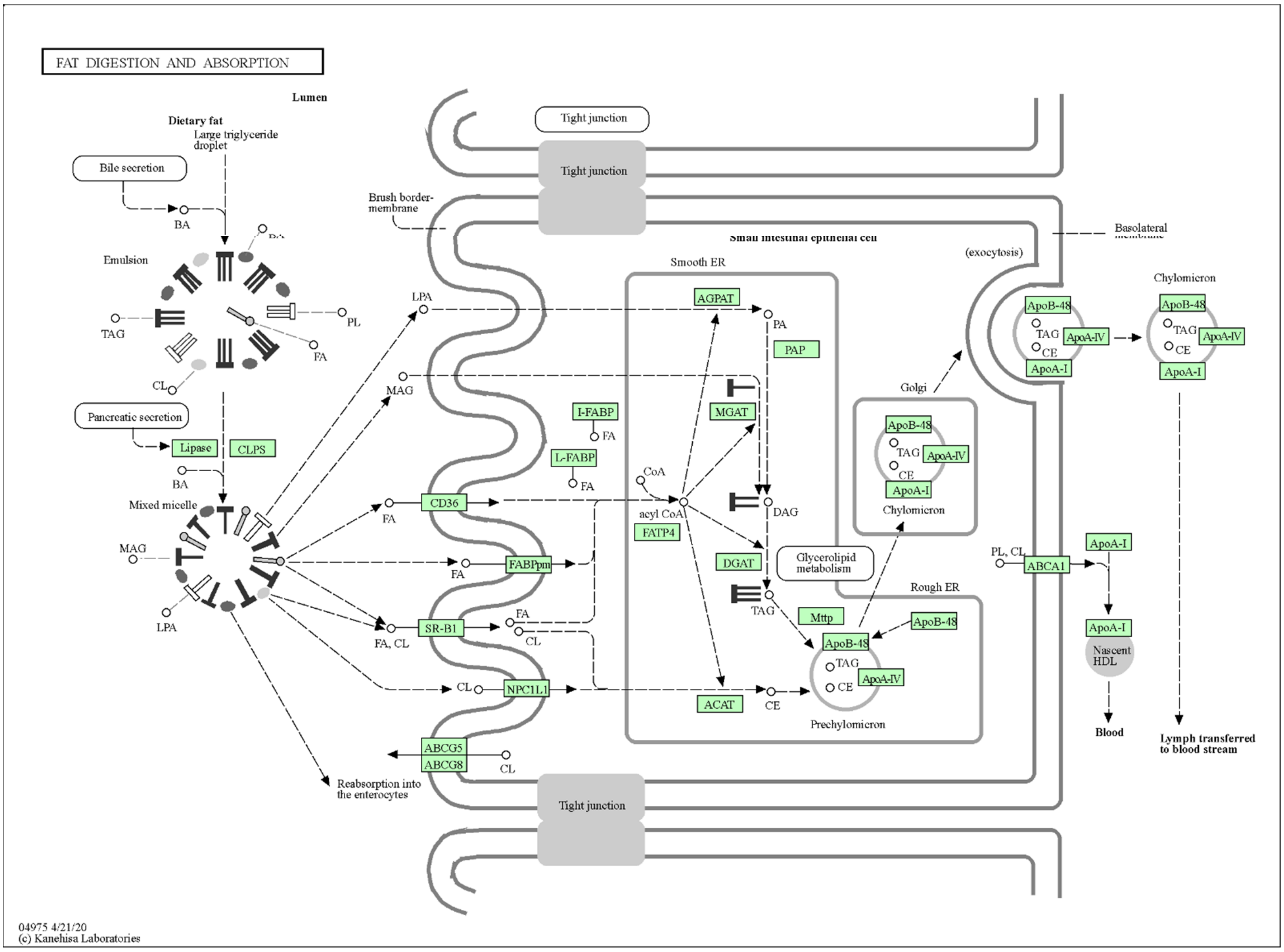

Figure S3 The fat digestion and absorption pathway. The figures are referenced from the KEGG PATHWAY Database (https://www.genome.jp/kegg/).

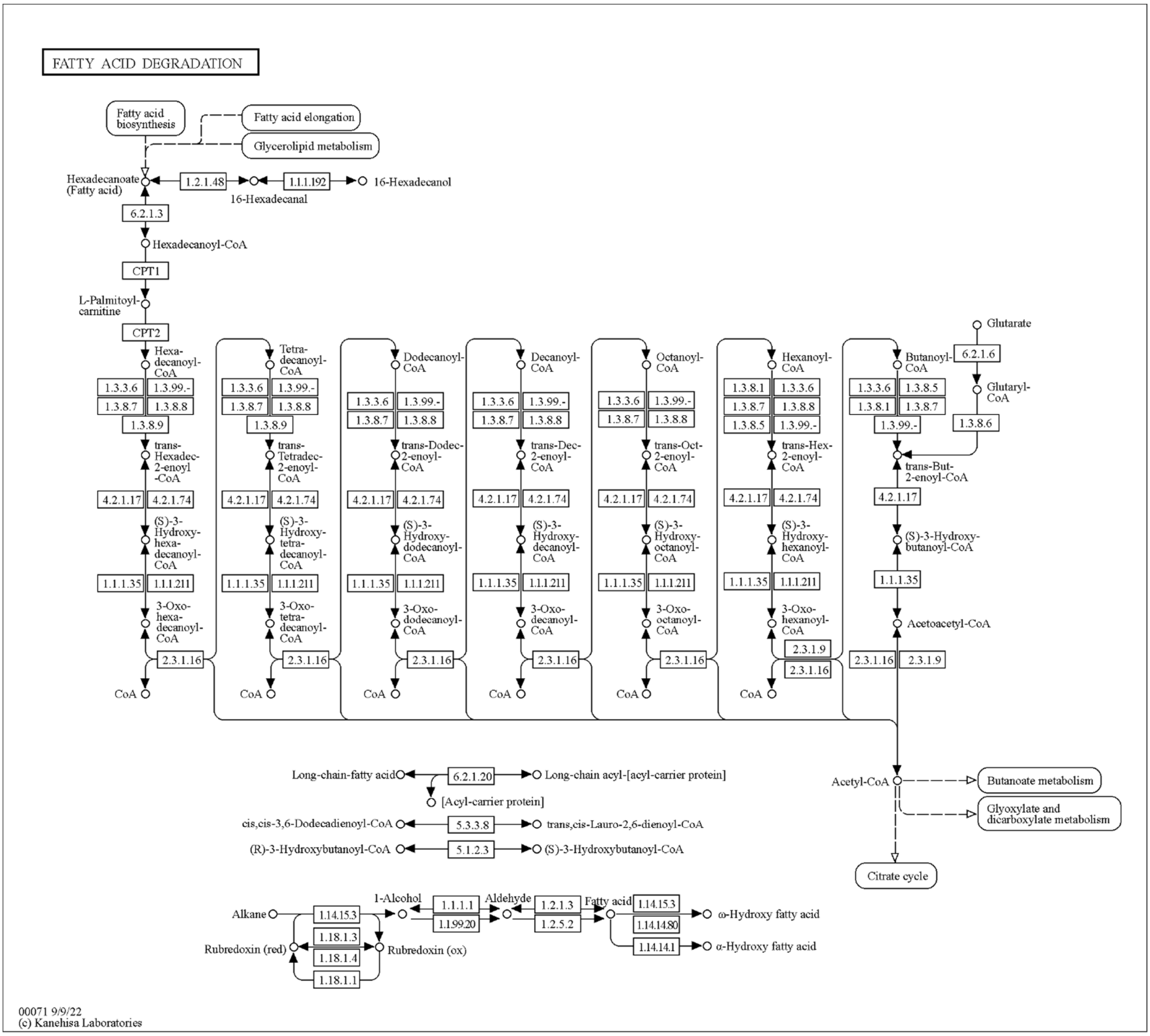

Figure S4 The fatty acid degradation pathway. The figures are referenced from the KEGG PATHWAY Database (https://www.genome.jp/kegg/).
